# Church Attendance: Its Influences on the Churchgoing Teenagers of the Redeemed Christian Church of God in the UK

**DOI:** 10.1007/s10943-025-02422-3

**Published:** 2025-09-01

**Authors:** Racheal Adebayo

**Affiliations:** Faculty of Health and Social Sciences, School of Applied Social Sciences, University of Bedfordshire, Luton, UK

**Keywords:** Teenager, Pentecostal, Church attendance, Black Majority Pentecostal Churches (BMPC), Redeemed Christian Church of God (RCCG)

## Abstract

This study examines the influence of church attendance on Pentecostal teenagers, particularly teenagers from the black-established Pentecostal Churches in the UK. It differs from previous research by focusing on church attendance rather than on religion and spirituality. This research was conducted among 27 teenagers, aged between 13 and 15 years old, who regularly attend the Redeemed Christian Church of God (RCCG), one of the Pentecostal denominations within the Black Majority Churches in Britain. The research was carried out as a qualitative study. The primary method of enquiry was face-to-face interviews. The data gathered were analysed using an interpretative phenomenological approach and thematic data analysis. The study argues that many Pentecostal teenagers in the UK, especially those who attend Black Majority Pentecostal Churches like the participants in this study, are not just actively engaged in religion and spirituality through the attendance of church services, as postulated by many academics from social sciences and religious studies backgrounds. Rather, church attendance has influenced the lives of youngsters in many positive ways. Critical analysis of the influence of church attendance among the interview participants falls into these themes: moral and social behaviours, personal and spiritual growth, and networking and corporate participation. In addition to presenting the empirical findings, this article compares such findings with the previous studies on the subject matter.

## Introduction

Several previous studies have reported a consistent influence of religion and spirituality among teenagers and children who profess the Christian faith. This includes the influence on their health and well-being, academic performance, moral and social behaviour, and social issues. While the influence of religion and spirituality on teenagers is numerous and diverse, studies on church attendance are limited. Despite the prevalent notion that teen church attendance is declining in the UK, the teenagers represented in this study are regular attendants at church services, attending at least twice every week. While youngsters can attend church events up to four times per week, depending on the activity they are involved in (Bible study, prayer meeting, choir rehearsal, and Sunday service), the average attendance for the recruited participants in this study is twice per week.

This study explores the influence of church attendance on the interview participants of this study.

### The Influence of Religion and Spirituality on Health and Well-Being

Research indicates that contemporary teenagers live in a society, especially in the West, exemplified by the cultural diversity of postmodernity or high modernity, escalating anxiety regarding risk, pervasive consumerism, fragmented families, and a significant transition towards gender equality (Singleton et al., 2004, p. 249). This has rendered it challenging for many teenagers to manage life circumstances, resulting in heightened instances of various anti-social behaviours and anxiety, which invariably detrimentally impacts their health. Nonetheless, research provides ample evidence that religion and spirituality equip teenagers with the ability to navigate adverse situations, providing them with coping mechanisms.

Evidence abounds in the literature that religion and spirituality equip teenagers with the skills to cope with unpleasant situations. For instance, Francis’s (2014) quantitative research shows a constant correlation between teenagers’ personal well-being and church attendance, with evidence showing that weekly churchgoer teenagers are more likely to believe that their lives are meaningful and worthwhile. Francis’s study supports a large body of other empirical research on the influence of religion and spirituality on teenagers. For instance, Werner and Smith (1992), and Crawford et al. (2006) claim that religion and spirituality help teenagers keep a positive attitude about life and find significance in challenging situations. Crawford, Wright, and Masten concluded that attending church services enables teenagers to cope in four important ways. First, it helps teenagers in developing attachment bonds; second, it provides open access to sources of social support; third, it guides teenagers’ behaviour and moral principles; and, finally, it provides possibilities for personal growth and development.

Furthermore, research affirms that religion and spirituality serve as a coping mechanism for dealing with adversity and hardship (Pargament, 1997; Francis, 2013)^.^ Pargament emphasised that attending church services fosters effective adaptive coping in times of adversity. As Francis (2014) reported, most of his churchgoing teenagers claimed that they received a high level of personal support from early postulated, close friends and their church community. They indicated that they were able to discuss their challenges openly with their parents, close friends, and even with their pastors when they needed help or empathy.

Additionally, explicit religion is arguably associated with an enhanced sense of purpose in life among adolescents who frequently attend church services (Dandelion and Collins-Mayo, 2010). As argued by Pearce et al. (2003); and Park (2007), religion and spirituality help teenagers cope with whatever life throws at them, such as depression, suicidal thoughts, and physical or mental problems, because it allows teenagers to develop meaning systems that sustain them when confronted with difficult life situations.

Consequently, teenagers who engage with religion and spirituality through frequent church attendance are more likely to effectively manage stress than their non-churchgoing peers.

### The Influence of Religion and Spirituality on Education and Academic Performance

Like the influence of religion and spirituality on the health and well-being of teenagers, academics have found that church attendance plays a positive role in the attitudes of teenagers towards school and academic attainment, including the way they view school environment, academic achievement, and their general attitude towards learning (Francis, 2013; Park, 2001). This is in addition to other factors, such as family upbringing, age, and gender, which have also been shown to contribute to how teenagers perceive the school environment and how well they perform academically (Johnson, 2008; Kim & Esquivel, 2011).

Research confirmed that teenagers who attend church regularly are more likely to see school as a happy place to be, feel positive about their teachers, and understand that school is preparing them for a better future. Johnson (2008), in his review of the scholarly literature, noted that 16 out of the 19 studies, he reviewed, demonstrated that church attendance had a positive influence on teenagers’ academic attainment. Johnson argued that Christian beliefs and the teenagers’ values, as well as family support, help them fulfil academic commitments, and achieve more, as they are more committed to education and earn higher grades than their peers who never attend church programmes.

Furthermore, Park and Bonner (2008) maintain that parental religious involvement, that is, where the whole family socialises through religious activities, is also associated with the higher academic performance of teenagers. Meanwhile, Park (2001) argued that teenagers’ commitment to church activities has a greater influence on their academic achievement than family variables such as socioeconomic status and parenting practices. Similarly, Regnerus and Elder (2003) confirmed that churchgoing teenagers who live in poorer areas are more likely to have high academic achievement than their peers who live in the same environment but do not attend church. Inversely, Francis (2014) found that most teenagers who attend church regularly are more likely to be anxious about their examination results and bullying at school than those who never attend church. This kind of anxiety often results in positive academic attainment and better support and coping mechanisms because churchgoing teenagers are more likely to seek help and academic support.

### The Influence of Religion and Spirituality on Moral and Social Behaviour

Parallel to the influence of religion and spirituality on teenagers’ health and well-being and education and academic achievements, studies have consistently proved that there is a positive relationship between church attendance of teenagers and their moral and social conduct. For instance, in their study, Furrow et al. (2004) claimed that church attendance gives teenagers a clearer sense of personal meaning and leads them to exhibit pro-social behaviour. Church attendance is associated with pro-social behaviour among teenagers, regardless of where they live (Francis & Mullen, 1993). Francis and Mullen claimed that there is a positive relationship between church attendance and a low rate of delinquency and violence, as religious involvement, demonstrated through church attendance, protects teenagers from negative behaviours by instilling moral values and attitudes, which produce positive behaviours. Arguably, churchgoing teenagers are less likely to abuse substances such as alcohol, cigarettes, and drugs, which can lead to anti-social behaviours.

Similarly, Donahue and Benson (1995) argued that teenagers who attend church are less inclined to participate in pre-marital sex and are more likely to join abstinence programmes because they tend to believe that sexual relations should only take place within marriage. As a result, they are less prone than their peers who do not attend church to engage in underage or pre-marital sexual acts. Additionally, they tend to have stronger opposition to abortion, homosexuality, and divorce compared to those who attend church occasionally or those who never go (Francis, 2013). Consequently, teenagers who regularly attend church are less likely to make choices that could be seen as socially or morally inappropriate.

### The Influence of Religion and Spirituality on Social Issues

In addition, studies have also established that churchgoing teenagers hold a positive opinion about the role of the church in society because they believe that the church must protect society. For example, in his report, Francis (2014) argued that both churchgoing and non-churchgoing teenagers maintain a high level of respect for the role of the church in society; nevertheless, churchgoing teenagers are more likely to be concerned about the effects of social media and technology on teenagers compared to the non-churchgoing teenagers. Specifically, they expressed concern about the number of inappropriate materials and images displayed in the media, whether on television, the internet, in magazines or on other public networks. Given there are limited studies on the influence of church attendance on teenagers, particularly those from Black ethnic backgrounds, this study was carried out to examine the impact of church attendance on teenagers from black majority Pentecostal churches (RCCG) in the UK.

## Materials and Methods

### Procedure

This study employs interpretive phenomenological approach to investigate the influence of church attendance on churchgoing teenagers from the Black majority Pentecostal organisation (RCCG). The interpretive phenomenological approach used in this study agrees in part with Smith’s (1996) statement that interpretive phenomenology is concerned with obtaining a description of the participants’ typical day-to-day lived experiences, such as workload, relationships with others, interactions, bodily experiences, and time experiences. I adopted the term “in part” because, rather than utilising an interpretive phenomenological technique to study every aspect of the participants’ lives, as Smith advocated, I employed the approach to investigate specific areas of the participants’ lives, namely their church attendance. The use of an interpretive phenomenological approach allows participants’ voices to be heard in scholarly study, as they describe and understand the realities of their church attendance.

The research questions addressed in this study include: *What is the influence of church attendance on black churchgoing teenagers in the UK?*

### Participants

The interview participants in this study comprised 27 teenagers of African origin, between the ages of 13 and 15, who attend the Redeemed Christian Church of God parishes in Leicester regularly, attending church services at least once a week. Researchers (Adebayo, 2019; Adedibu, 2016) described the RCCG, an African-established Pentecostal denomination, as the fastest-growing denomination within the BMPC in Britain. Arguably, one of the contributing factors to the rapid growth of the RCCG is the large number of young people who attend the denomination (Burgess, Knibbe, & Quaas, 2010; Hunt, 2002). Burgess, Knibbe, and Quaas (2010) maintained that the RCCG in Britain has the highest proportion of young people in attendance in comparison with other early-established Pentecostal organisations in the UK. The participants in this study were recruited through purposive sampling after obtaining approval from the Association of Research Ethics Committees. Additionally, consensus from the participants’ gatekeepers was obtained as the participants were considered minors at the time of data collection. The participants’ consent was also obtained before data collection.

### Instrument

To investigate this topic, 27 participants were individually interviewed using a semi-structured approach. The topic and structure of the interview questions provided a framework for the subsequent data analysis (Smith, 1996). The interviews were primarily conducted at the participants’ parishes, except a few times when they were held in the participants’ homes at their request. In addition to the audio tape used for recording the interviews, field notes and a notepad were also used to document some actions that may be difficult to capture electronically. For example, body language such as mood and pauses, which are very important at the time of analysis to gauge, not only what was said, but how it was said. To protect the participants’ privacy, pseudonyms were utilised for the transcriptions and analysis.

#### Analysis

Data collected for this study were analysed using interpretative phenomenological analysis (IPA). According to Smith et al. (2009), the most crucial aspect of conducting an IPA analysis is making sense of the research participants’ experiences. Furthermore, Kay and Kingston (2002) suggest that an IPA is “particularly suitable where one is interested in complexity or process, or where an issue is personal” (p.171) and where a deeper, personalised examination is required, such as in this present study.

The interview process adopted in this study include one-to-one interview with the participant, who had been purposively recruited. Following the interviews, all audio-taped data were manually transcribed into a line-numbered transcript. Each transcript was coded, and the participants’ comments were compared to all data. Coding entails “careful reading and re-reading of the data” (Rice & Ezzy, 1999, p. 258) to find and document initial themes and concepts that are common and related to the research question. A list of preliminary codes was created using the study question, transcripts, and interview notes. The codes were subsequently categorised based on their similarities and differences. The classification procedure resulted in thematic findings about the influence of church attendance among the interviewed participants.

## Findings and Discussion

Although the impact of church attendance on teenagers varies based on factors such as age, background, personality, and frequency of attendance, this section focuses on the main influence identified by the study participants. This influence encompasses moral and social support, personal and spiritual growth, and networking and communal participation. The church fosters these aspects through corporate programmes and activities, including prayer, Bible study, and evangelism, along with networking and community involvement in denominational events.

### The Influence of Church Attendance on Moral and Social Behaviour

The majority of participants in this study specified that church attendance was extremely crucial to them. They claimed that their attendance enhanced their moral and spiritual growth. This is evident in how frequently they reported attending church programmes, with the majority going more than once a week. According to these teenagers, the amount of counselling and coaching provided by their peers and adults in their Parish has a substantial impact on their moral and spiritual lives. One of the participants said: “If you are not attending church regularly, you are the one missing out on getting to know God better and building a relationship with Him because when you go to church, you will have people, who will encourage you” (Joseph, 14).

Joseph also stated:

Hmm, there are lots of decisions that you [I] wouldn’t make as a Christian, but if you [I] weren’t a Christian, you [I] would make wrong decisions. But attending church programmes has helped me to know which decisions are good for me. I know that going to school is important, and getting good grades is also important.

Joseph then declared, “If not for the church, I would have been hanging out with the wrong crowd”. According to Joseph and other participants who share his viewpoint, the support they receive in the church motivated them to make great decisions about their lives, such as having a positive attitude towards education and achieving good results on their examinations. They claimed that the moral and social support they received from the church empowered them to focus on important and purposeful areas of life.

Amos (15) additionally described how church attendance influenced his moral and social life, particularly his ability to make decisions: “Well, when you want to do certain things, you don’t realise maybe it’s bad or not, but after you attend the church. Your church attendance teaches you that such behaviour is bad for a Christian”. When asked to clarify what he meant by “certain things”, Amos replied:

“Maybe you find people using methods that, as a Christian, you consider not good to get money, like hacking, tricking, or something. And you would have wanted to join in since you see them enjoying their money. After seeing the outcome, you realise that your church attendance was essential in keeping you from doing it”.

Moses (15) reaffirmed the preceding statement, claiming that having a network of peers in church for support has positively impacted his life in general, and, specifically, how he approaches religious and spiritual issues because church attendance has allowed him to learn from the experiences of others, including his peers. He continued, “Because there are many teenagers and friends who, you know, they’ve been through the same thing you’re going through, and they can relate to it better. They help you stay focused and so on”. Moses stated that attending church has given him the capacity to relate to a group of peers who share his values, viewpoints, and challenges on life’s issues and situations, as well as to seek help when needed.

Consequently, church attendance enables young people to develop social support networks with both peers and adults. The support they receive increases their self-esteem and confidence in their talents. It also provides them hope for the future, knowing that they are not alone in their struggles and can easily find others who can relate to their experiences. They also stated that attending church helps them meet people who can serve as mentors and role models. This indicates how frequent church attendance can help youngsters.

The findings on the moral and social support that the participants in this study revealed are consistent with earlier research (Park & Bonner, 2008; Smith & Snell, 2009) that showed that teenagers’ academic performance is positively impacted by church attendance. Park and Bonner, for example, contended that church attendance, especially when parents attend, had a favourable impact on teenagers’ academic performance since it would have inspired both parents and teenagers to value education and academic success. The findings of this study are also in alignment with Francis’ (2013a, 2013b) research, which found a link between young people’s sense of purpose in life and church attendance. Francis asserted that teenagers who attend church have peers and adults to help them navigate this time, which makes them less anxious about their relationships with others. However, others in their age group who do not attend church services may lack this guidance. In other words, church attendance enables young people to look forward to a brighter future, reducing the social issues that other youths may be facing, or at least how the social issues may impact them. Furthermore, it allows them to socialise with friends who share their interests, which protects them from negative peer pressure, which can lead to unwanted actions. Church attendance also allows teenagers to form strong, long-term relationships with adult and peer role models, especially parish leaders (Yust, Johnson, and Sandy, 2006). Similarly, Hunt’s (2002) research participants cited the teachings and mentoring they received from their various congregations as motivators for regular church attendance. For example, when Hunt’s participants were asked what they enjoyed best about visiting their Church denomination, they said that the lessons on realising one’s potential stood out to them since they dealt with practical aspects of life. It also emphasises that the Church values its members’ potential, whether spiritual, emotional, or professional, rather than just providing them with spiritual sanctuary.

Inversely, the study’s participants claimed that church attendance has no tangible or financial benefits for them. This contradicts Garrald’s (2009) investigation, which found that one of the advantages of church attendance among black people in the UK is the financial help provided by churches to its members. While this study does not rule out the possibility that youngsters in BMPCs received financial or material support from the church at some point, none of the interviewees confirmed this. Rather, they all claimed that the church’s primary influence on them is the spiritual and moral support they receive through mentorship, counselling, and coaching from other church members, including their peers and teachers. This agrees with the findings of Hunt (2002) that material or financial incentives may not always act as motivation for church attendance among teenagers in the BMPCS, as indicated by Garrald (2009).

### The Influence of Church Attendance on Personal and Spiritual Growth

Another influence of church attendance mentioned in this study is personal and spiritual growth. A substantial number of interview participants in this study claimed that church attendance and participation improved their personal and spiritual development. This includes their prayer lives and spiritual journeys, as demonstrated by their devotion to Bible study and evangelism. For example, Joy (13), when asked about the church’s influence on her, responded that:

[The] church has influenced me because we have Bible studies in which we choose a specific book or topic and examine the lessons from it. We also hold prayer gatherings where we all get together to pray. Sometimes we were assigned to lead the entire church in Bible study and prayer. I believe this has helped me.

Although Joy did not specify how leading the Bible studies and prayer sessions benefited her, it is assumed that such activities taught her how to prepare and deliver a presentation in front of small and large groups of people, as anyone who leads a Bible study or a prayer meeting is expected to be well-prepared for the task. Joy would also have learnt about time management, as each of the two programmes she described had a specific amount of time assigned to it that she would not want to exceed.

Similarly, Victoria (14) said that studying the Bible had a major impact on her. This is how she articulated it:

When I read the Bible, I see the consequences that God imposes on individuals who have sinned in the past. Then I see other people my age, such as pregnant women who smoke, and I realise how disappointed God is in them because God [sacrificed] his son so we might live, and we’re just taking advantage. So, I believe that reading the Bible and going to church are making me feel better about myself. [Be]cause generally, when I’m upset with my mother and go to church, I alter my attitude because I believe God placed me here for a reason, and I don’t want to fail Him.

One could argue that Victoria’s response demonstrates the power of the Word (the Bible) to change people’s lives. Reading the Bible, either alone or with other church members, influenced her behaviour. She conveyed her thanks for the change. She also acknowledged that God has a purpose for her life, and she did not want to compromise it by becoming upset or engaging in “sinful” behaviour. Nonetheless, Victoria expressed regret that some young people do not have the opportunity to learn what she knows since they do not attend church programmes. As a result, some of them started smoking and drinking, and others were pregnant as teenagers.

Joy (13) agreed with Victoria, claiming that Bible reading during church programmes and at home had taught her resilience. And that visiting church to hear God’s word and receive encouragement from other churchgoers had enabled her to become strong. She believed that these lessons and encouragements have helped her develop a personal relationship with God. She mentioned that she has grown from someone who is continuously receiving to someone who can stand among others and teach as an assistant Sunday school teacher. This was how Joy described her experience.

They [adults and peers] motivate me to be more confident and to do better things, such as study the Bible and pray; they may also teach you things that others may not be able to. They [her Sunday school teachers] also offer me the opportunity to serve as a teaching assistant once a month in the younger class, which I believe will help me in the future if I decide to pursue a teaching profession. (Joy, 13)

Similarly, Angie (13) mentioned that there was a lady in her old church who was quite passionate about Christianity.

"When she was preaching, she would start speaking in tongues. She would cry as she started lecturing and she made me think… when I grow up, I want to spread the Word of God and be like her, even if it starts with my brother.’ I will call my brother and say, Come, let me teach you, … let me see how much you know about God’s word."

Likewise, Zoe (15) noted, “Ever since I started going to church, I’ve learned new things about God and Jesus. As a result of what I’ve learned at church, my faith in God has grown stronger and I have been preaching to others”. The findings of this study’s participants align with Francis and Evans (1996), indicating that church attendance improves the personal prayer practices of young people, hence assisting them in discovering their life’s purpose.

As demonstrated by this study, church attendance encouraged individuals to be courageous in representing the church in civic initiatives, which includes participating in The Street Fairs, where church members, including the participants in this study, can speak one-on-one with people in the community about their faith, evangelise, or collaborate with other church denominations and Parishes to serve the community. Tammy (15) mentioned that church attendance influences her evangelism. According to her, “The Street Fairs are when we evangelise and reach out to everyone, especially when the youths have to reach out to the young ones and teens. I can listen to their perspectives on God and discuss my own as well”. These participants argued that representing their Parishes in different corporate programmes helps build their confidence in public speaking and trains them to be able to defend their faith as they meet with peers, whether from school, other Parishes, different church denominations, or those that are not of the same faith as they. In agreement with Kim and Esquivel’s (2011) research, this present study demonstrates that engagement in social and civic activities through church programmes, such as evangelism, mission projects, tutoring, and helping people in the community, helps teenagers to find meaning as they extend themselves beyond narrow self-consciousness and broaden their horizons.

Furthermore, interviewees claimed that having a separate church service for teenagers in their respective Parishes influences their personal and spiritual growth because they can express themselves within a circle of their peers, discussing issues more relevant to their age group, rather than being asked to join the larger church group. They also mentioned that having a specific age-appropriate curriculum developed for them is beneficial in meeting their requirements because it is suited to their level of comprehension. Likewise, the majority of the participants agreed that the teaching techniques enabled them to apply theory to practice, as did practical demonstrations offered by the teachers. For example, Eniola (13) admitted that a distinct church programme for the youth in her parish allows her to relate with people who understand her and can help her spiritual growth as she navigates her teenage life. This is how she described it:

… I go church every Sunday because we [teenagers] have a separate service from the adults. In [the] teen church, we typically discuss personal experiences and spiritual matters. It depends on how we feel, indeed. And I can express my feelings and what I’m going through with individuals that understand.

Similarly, most of the participants acknowledged that they benefited from church programmes and activities, such as the annual Bible club, where children from other churches are invited to take part in a whole day of creative Bible lessons and Christian-related activities, such as organising a movie, quizzes, debates, and so on. They also mentioned Youth Week, which is a programme that takes place once a year in all the RCCG Parishes at different times. During this programme, the youth are given the keys to the church for a week. They are the ones who control every activity of the church, including all the weekday programmes and Sunday services, which include preaching. The youths are allowed to bring whatever creativity they want into the programme during this week, as long as they are in line with the teachings of the Bible and the doctrine of the Church. Many who participated in this programme maintained that it enhanced their relationship with God. For example, Lucy (13) stated, “I like it when, every year, we [church] have a date when the children take over the service, so they do praise and worship, they do the sermon, and I think they [church] should do that more often because it makes us [teenagers] connect more with God and worship”.

The participants also stated that they benefited from the youth-organised song festival and entrepreneurship, where they are given a free hand to organise programmes, such as the praise festival or charity work, on behalf of the church. They are tasked with raising funds for this programme through entrepreneurship, such as selling tickets, cakes, or other things that could generate money. According to them, these programmes have helped them with organisation and leadership skills. The programmes described above encompass ages that are not covered by this study because the RCCG’s youth church is for ages 11–16, but the bulk of the responsibility falls on 13–15 years age range. This study’s target audience (13–15) is significant because it is referred to as a “forgotten” age, because less research has been conducted on it (Francis 2014). In addition, this age group can be tough for parents and society because of the advent of puberty, increased peer influence, and the transition to more complex academic and social settings. It is also a developmental stage in which teenagers recognise the importance of having a positive relationship with themselves and the world around them, prompting them to engage with religion and spirituality more than younger children (Lerner et al., 2011). This age bracket differs from 16 to 19 years old, who are also classified as teenagers, in that teenagers in the targeted age continue to live with their parents, whereas teenagers in the 16–19 age bracket have gained some independence from their parents, either through a career or higher and further education.

This finding is consistent with the reports of Blakeney and Blakeney (2006) and Johnson (2008), which demonstrate that a sense of social responsibility and care for others can help teenagers improve their well-being and reduce anti-social behaviours. Therefore, church attendance provides an avenue for teenagers to learn about how to increase the scope of their spirituality, build relationships with others, and engage in civic activities (Burgess, 2009). Having discussed the influence of church attendance on the personal and spiritual growth of the participants in this study, the next section discusses the influence of church attendance on networking and communal participation of the participants.

### The Influence of Church Attendance on Networking and Communal Participation

In addition to the previously mentioned influence of church attendance, interview participants stated that it allows them to network with people from various RCCG Parishes as well as participate in the Church’s wider corporate initiatives. For example, they mentioned the Festival of Life as one of their favourite opportunities for networking and corporate participation. Festival of Life (FOL) is a bi-annual programme organised by RCCG executives in the UK for all RCCG members and some Christians from other denominations. It is an extension of the Holy Ghost Festival, which takes place every month in Nigeria. During this programme, a guest minister and the General Overseer of the RCCG (Pastor E. A. Adeboye, popularly known as “Daddy GO”) usually lead the words of exhortation. On numerous occasions, people have testified to God’s power for healing and deliverance from demonic attacks, debts, and childlessness, and some have come to praise God for various blessings given to them. Pastor Adeboye’s prayers of deliverance and blessings are frequently featured in the concluding half of the presentation. Many participants claimed that the General Overseer’s prayers over them were answered. The RCCG, UK always encourages its members to attend the programme as a family. As a result, the majority of teenagers would have visited the FOL with their parents at some point.

One example of the participants, who claimed that the FOL has a great influence on her life, is Natalia (13). According to Natalia:

Erm, I like Festival of Life because is like the whole Redeemed has to come together. Like a lot of churches [Parishes] just come down to worship God and have a good time, and just thank Him for everything, and share their testimonies. We know you go there believing that He [God] answers all of our prayers. And it’s just a good thing in my life [because] it brings unity. I can see people from different cities and talk to them freely. I often make new friends with people I may not see again.

Natalia also stated, “I love the praise and worship section at the FOL. It’s just a good way to connect to God. And I like the interactive session we have to connect with other people and just discuss life in general and make friends”. As stated by the participants, the attendance at the Festival of Life allows them to connect with other teenagers from different Parishes, and it provides an occasion for making new friends among a group of people who share the same faith and philosophical views with them.

However, despite all the plaudits about the influence of church attendance among teenagers, especially the influence of older church members, a few of the participants, Grace (15) and Precious (14), show an exception. Grace conceded that “[having] like different classes where we learn stuff about Christian teenagers contributed to my religion and spirituality”. Nonetheless, she claims that the age gap between the teachers and the teenager students frequently impeded her ability to connect with the curriculum delivered in class. She observed that teachers’ personal experiences, which they typically use when teaching, are vastly different from hers. Specifically, the teachers had grown up in Nigeria rather than the UK, where she and many other participants are growing. As a result, Grace felt that the lessons, in most situations, do not serve a constructive purpose in her understanding of religion and spirituality. Grace assumed that these teachers do not understand what she and the other participants experience as teens in the UK. She also bemoaned about the lack of teenagers her age with whom she might identify in the Parish she attends.

According to her:

In our church, there are not many teenagers and the teachers are not our age. As a result of our generational differences, they are unable to comprehend the challenges we face. They would most likely not have been aware or have experienced it because they grew up in a different era, and the most of them did not grow up in England. As a result, they have had a different experience in life.

Grace was not alone in believing that the generational and cultural gaps between the teachers and teenagers in the church had not benefitted the latter. Likewise, Precious (14) argued:

I don’t think the older people in my church [including the teachers] contributed to my religion and spirituality. Erm, because most of them are like parents. I wouldn’t say old, but they’re way older than me. So, I wouldn’t be able to connect with them or discuss what I’m going through with them.

Grace and Precious believe that the capacity to connect is critical for teens to have a positive influence, and they believe that their churches are failing in this regard. They expect the church to provide a way for them to connect, but it has not done so through its teaching. As a result, they declared that the church had failed to meet a few of their requirements. Grace and Precious both thought that their Parishes had not done enough to recruit more teenagers to the parish and that adults had not been able to bridge the age gap and social contexts that may have existed between them and these young people. Consequently, they believe that the benefits of attending church services have not been completely achieved. In the light of these teens’ opinions, this study proposes that the RCCG leadership in Leicester collaborate and design a programme, even if it is only once a month, in which all RCCG youngsters, without exception, can join together for fellowship.

## Research Limitations

This study employs an Interpretative Phenomenological Approach, which has both advantages and disadvantages. It acquired an interview strategy, which prioritises how participants chose to characterise their experience over observation of the experience itself. In an IPA method, the participants’ ability to communicate and make sense of their experiences is critical. However, this recounting of prior experiences was based on their ability to recall events and experiences, which may not be entirely factual, as well as subjective.

Another weakness of this study is the setting. The study’s participants are all Black African teenagers aged 13–15, primarily from Nigeria, with a few from Kenya and Ghana. It is assumed that findings among young people of different denominations will yield diverse outcomes. Consequently, future research could build on the knowledge provided by this study by exploring the religion and spirituality of teenagers from various African ethnic groups or age categories. Additionally, all 27 participants in this study are members of the same denomination, the Redeemed Christian Church of God. Furthermore, all 27 participants belong to the same denomination: the Redeemed Christian Church of God. Arguably, the teachings and beliefs of this denomination may have influenced the church attendance experience documented in this study.

Furthermore, the study takes place in Leicestershire, which is located in England’s East Midlands. While this setting has an advantage in that the RCCG grew rapidly in this location more than any other place in the UK, apart from London, it is crucial to highlight that findings from other contexts may differ from those reported in this study. Nonetheless, the findings in this context can be used as a guide for comparable investigations in different settings. Equally, the author wishes to conduct a similar study from the perspective of teenagers from a different context and background.

## Conclusion and Recommendation

The findings of this study are consistent with the previous research on the influence of church attendance. The majority of participants of this study indicated that church attendance has positive impacts on them in three areas: moral and social behaviours, personal and spiritual growth, as well as networking and corporate participation, albeit the level of influence differs from one teenager to another. The study concludes that coaching and mentorship from adult church members because of regular church attendance influence participants’ moral and social behaviours, shaping their decision-making and attitudes towards educational achievement and engagement. Church attendance is also believed to benefit participants’ personal and spiritual growth by enabling them to cultivate personal relationships with God through independent and corporate prayers, Bible study, evangelism, community service, and entrepreneurship. Furthermore, the study demonstrates how regular church attendance, as well as participation in programmes organised for the entire RCCG in the UK, facilitates opportunities for fellowship, networking, and communal engagement among a large group of teenagers, resulting in teenagers’ unity and connections.

Finally, while this study was conducted in the UK among a particular ethnic group and church denomination, which precludes generalisation, the findings provide an intuitive picture of how church attendance affects teenagers, which can be applied to other scenarios with teenagers in a similar age range.

Because of the scarcity of studies focusing on teenagers from ethnic minority groups in the UK, more research is required. It is especially significant because research has revealed that these groups account for the vast majority of youngsters who continue to attend church services. This will allow for comparisons of what needs to be done in other churches where youngsters have ceased attending services. To get a comprehensive picture of the impact of church attendance on young people in the UK, similar research from several denominations and/or age groups is recommended.

## Appendix 1

See Table 1.

**Table 1:** Demography of the participants

	Name	Sex	Age	Parish	School
1	Amos	M	15	Green pasture	Mainstream school
2	Angie	F	13	King of Glory	Mainstream school
3	Anthony	M	13	Amazing Grace	Faith school
4	Faith	F	13	City of Favour	Mainstream school
5	Felicia	F	13	Amazing Grace	Mainstream school
6	Florence	F	13	City of Favour	Faith school
7	Georgina	F	15	Amazing Grace	Faith school
8	Goodness	M	13	Covenant of Grace	Faith school
9	Grace	F	15	Amazing Grace	Faith school
10	Jane	F	13	Amazing Grace	Faith school
11	Joe	M	15	Amazing Grace	Faith school
12	Joseph	M	14	City of favour	Mainstream school
13	Joy	F	13	Amazing Grace	Faith school
14	Jude	M	13	City of Favour	Faith school
15	Justin	M	13	Amazing Grace	Faith school
16	Lucy	F	13	City of favour	Faith school
17	Mary	M	13	City of Favour	Faith school
18	Matthew	M	14	City of Favour	Mainstream school
19	Mercy	F	13	City of Favour	Mainstream school
20	Moses	M	15	City of favour	Mainstream school
21	Natalia	F	15	King of Glory	Faith school
22	Oscar	M	13	Amazing Grace	Faith school
23	Peace	M	13	City of Favour	Mainstream school
24	Precious	F	14	Green Pasture	Mainstream school
25	Shimon	M	14	Amazing Grace	Faith school
26	Victoria	F	14	Green pasture	Mainstream school
27	Zoe	F	15	City of Favour	Faith school

## Sample of interview questions on the influence of church attendance

### Participant’s Personal Information

Tell me about yourself, your name, age, church, and school.

How long have you been in Leicester?

Research Question: What are the impacts of church attendance on the black churchgoing teenagers in Leicester?

## Interview Questions

There have been various studies in this country on how young people are leaving the traditional (orthodoxy) churches to join other (Pentecostal) churches, and some have even stopped going to church completely. I want to know your thoughts on church attendance.

- Have you always been a member of the RCCG? When did you join the church?
- How often do you go to church, and what sort of activities do you do in the church?
- What aspect of the service or programme do you enjoy most in the church? Why?
- How do you think Church attendance has influenced you?

You remember that in the RCCG, prayer, Bible study, and Christian values (morality and holy living) are emphasised a lot.

- I would like to know what your experiences are in these areas. How often do you pray?
- When do you pray? For example, personal time, family time, or corporate time in the church? How often? Why do you pray?
- Do you study your Bible? How? Why? How often?
- As a young person, it is possible to make choices, both good and not so good. Could you tell me about your experience of the time you made some good choices and what influenced you to make these choices?
